# Honing the Priorities and Making the Investment Case for Global Health

**DOI:** 10.1371/journal.pbio.1002376

**Published:** 2016-03-02

**Authors:** Trevor Mundel

**Affiliations:** Global Health Division, Bill & Melinda Gates Foundation, Seattle, Washington, United States of America

## Abstract

In the aftermath of the Ebola crisis, the global health community has a unique opportunity to reflect on the lessons learned and apply them to prepare the world for the next crisis. Part of that preparation will entail knowing, with greater precision, what the scale and scope of our specific global health challenges are and what resources are needed to address them. However, how can we know the magnitude of the challenge, and what resources are needed without knowing the current status of the world through accurate primary data? Once we know the current status, how can we decide on an intervention today with a predicted impact decades out if we cannot project into that future? Making a case for more investments will require not just better data generation and sharing but a whole new level of sophistication in our analytical capability—a fundamental shift in our thinking to set expectations to match the reality. In this current status of a distributed world, being transparent with our assumptions and specific with the case for investing in global health is a powerful approach to finding solutions to the problems that have plagued us for centuries.

## Abstract 2

We need an investment strategy that goes beyond simply making passionate pleas to control and eliminate the preventable diseases that affect millions around the world.

When we have proactively set our sights on large and defined obstacles to human wellness, the global health community has been able to chart a course toward lasting, widespread impact. However, few would argue that the global health community’s response to Ebola—while ultimately effective—was the optimal way to anticipate and address a global health crisis. Comprehensive analyses have been conducted on what worked well and what didn’t [1]. Despite all the failings that led to over 11,000 deaths and an estimated US$1.6 billion in costs to the economy in Guinea, Sierra Leone, and Liberia, the global community did come together and help turn the tide against the epidemic—albeit more slowly than what could have been possible with a better-prepared world [2,3]. Major funding commitments were made when the reality and urgency of the epidemic became evident [4]. Another point that may not be widely known is that the private sector responded to the challenge by directing significant resources to develop vaccines, drugs, and diagnostics at an unprecedented pace. As a result, we now have four vaccine candidates, three therapeutics in Phase III clinical trials, and six diagnostics authorized for emergency use by WHO [5].

At the turn of the millennium, the global community sought to address the far more complex problem of vaccination coverage. In 2000, the glaring disparity in vaccine access between wealthy and developing nations led to the formation of Gavi, the Vaccine Alliance [6]. After 15 years, Gavi has helped create a roadmap for countries to ramp up their immunization programs—reaching nearly half a billion additional children with vaccines [7]. Through its multisector partnership, Gavi not only addressed the huge challenge of improving childhood vaccination coverage, but it also provided certainty to the private sector, encouraging it to manufacture products for developing country markets and to make them affordable. In 2015, donors came together again and made US$7.5 billion in pledges, the largest ever financial commitment to support childhood immunization [8].

We can find a comparable example in the sobering problem of tuberculosis (TB), in which the battle is being fought with a decades-old and unwieldy six-month treatment regimen of diminishing efficacy due to multidrug resistance. In 2013, TB made an estimated 9 million people sick, and 1.5 million people died from the disease [9]. However, the fight against TB is being reinvigorated. The TB Drug Accelerator (TBDA) is a groundbreaking partnership among eight pharmaceutical companies, seven research institutions, and a product development partnership funded by the Bill & Melinda Gates Foundation [10]. By driving collaboration and data sharing atypical of its partners, the TBDA’s overall goal is to create a new TB drug regimen that cures patients in only one month, replacing the outmoded intervention we have today. While the structure and purpose of the TBDA took rigorous iteration to get where it is today, the data and expertise shared among its partners has already identified compounds that could potentially lead to a more effective treatment.

Although it might seem that the motivations behind the investments in these three cases are different—a potential regional or global health catastrophe in the case of Ebola, a humanitarian imperative underlying Gavi, and a dual global drug resistance threat and humanitarian basis for the TBDA—there is a common thread. These examples show that when there is imminent and clear need, we have been able to mobilize resources and construct creative partnerships to create an impact. Summers et al. make the compelling case for investing in global health by showing the general economic benefit of those investments [11]. Similarly, others have claimed a substantial return on investment in specific areas of health science as a motivation for future investments [12]. These cases are fairly general in their content, and we see modern investors in global health (whether countries or philanthropists) as being much more demanding in terms of wanting to know exactly how their resources are deployed and the impact that could be expected.

At the Bill & Melinda Gates Foundation, we are exploring a set of approaches that start with our current (and improving) knowledge of the state of the burden of diseases relevant to low- and middle-income countries (LMICs) and comparing the potential interventions we have to reduce this burden along a number of dimensions. The ultimate objective is to arrive at a view of the actionable priorities that we can support at any one time in order to maximize our impact on health and wellbeing in communities with the highest burden. This approach has some parallels with portfolio analysis in the biopharmaceutical industry [13], but the very sparse and poor quality of the underlying primary data in global health means that, at best, we can rely on this as a rough guide and a mechanism for exposing outliers in cost, effectiveness, and impact (Fig 1). This approach provides a framework for comparison across diverse categories through a metric that is understandable. More importantly, it forces us to state our assumptions explicitly for debate and reconciliation. However, we also need to be cautious about any notion that the complex sociopolitical environments we work in and the fluctuating humanitarian crises that arise can ever be reduced to simple algorithms for decision-making.

**Fig 1:**
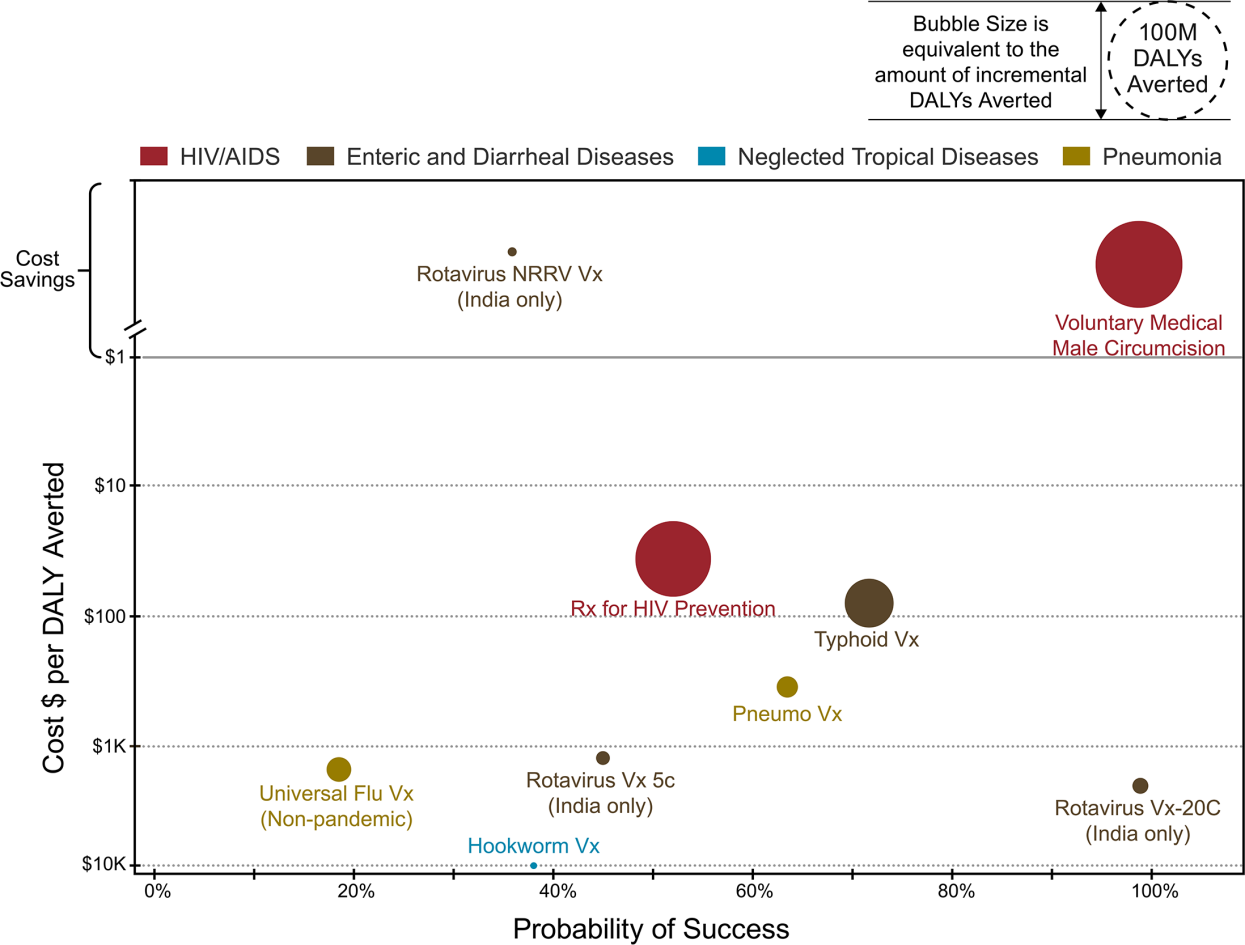
Portfolio analysis for global health impact. Cost per disability-adjusted life year (DALY) averted is the incremental cost to deliver incremental DALY savings versus only the standard of care. Probability of success is the estimate of probability of technical and regulatory success (PTRS) informed by industry benchmarks and expert opinion. NRRV: Non-replicating rotavirus vaccine. Both the cost and the probability of success are dynamic values and subject to change with information that is constantly evolving.

Although our understanding of the burden of disease has improved tremendously at a national and subnational level for important pathogens, as evidenced by a recent integration of our knowledge of the spatial distribution of the risk of malaria in sub-Saharan Africa [14], we need to invest much more heavily in obtaining better primary data. We therefore recently launched CHAMPS, the Child Health and Mortality Prevention Surveillance Network [15]. CHAMPS will be a network of disease surveillance sites in LMICs that will help gather accurate data about how, where, and why children are getting sick and dying. For the first time in history, pathology-based surveillance will be used to track the causes of childhood mortality, complementing and improving upon existing cause-of-death information from verbal autopsy surveys and vital statistics. Through geospatial modeling and mapping, these new surveillance data will provide an increasingly broad and accurate picture to guide more effective use of the scarce resources for prevention and treatment.

Improved data can also help drive progress against less familiar health challenges such as neglected tropical diseases (NTDs) [16]. Until recently, little was known about the geographical distribution of NTDs. Because of weak surveillance systems, the scarcity of geospatial mapping was greatest in sub-Saharan Africa, which has hampered deployment of effective programs. To address this, a WHO African Region-led effort has conducted thousands of field surveys using mobile phone data capture to complete the picture of NTDs across Africa. In addition to guiding disease control efforts, such as targeting of mass drug administrations only to places that need them, this mapping provides, for the first time, the necessary central database to allow analysis of program performance and to make projections of likely outcomes, including the probability of disease elimination.

The framework we use to evaluate this data has a few simple dimensions: cost per disability-adjusted life year (DALY) averted, probability of technical success, and, at a more strategic level, whether our resources fill a real gap in the funding landscape (Fig 1). There are many alternative metrics, but we have chosen this scheme for its simplicity and augment it with additional analyses when these make sense. An important part of the framework related to work that might only be completed well into the future is understanding the spectrum of potential trajectories for future disease burden. Thus, forecasting becomes an essential element of decision-making; for this, we need to go beyond only linearly extrapolating future outcomes based on past trends. Much more sophisticated forecasting that integrates all significant covariates of the main outcome is becoming available [17] and will be increasingly useful for decision-making and the longitudinal evaluation of projects to assess whether interventions are shifting the envelope of outcomes in a positive direction.

The system described above, which is already in use across parts of our global health portfolio, makes us optimistic that we can, in the near future, expand this approach to the entire portfolio and arrive at a more systematic way of understanding the inherent values and risks of a given intervention. This will also give us a clear picture of the huge and urgent problems in global health, paired with more deeply evaluated and cost-specific solutions, as well as a forecast of the negative consequences of inaction. Much as the world, or parts thereof, were mobilized by the Ebola crisis, the childhood vaccination gap, and the TB epidemic, we would then have maximized the likelihood of accessing new resources for potential solutions to ongoing global health crises.

## Abbreviations

CHAMPS: Child Health and Mortality Prevention Surveillance Network
DALY: disability-adjusted life year
LMIC: low- and middle-income countries
NRRV: non-replicating rotavirus vaccine
NTD: neglected tropical disease
PTRS: probability of technical and regulatory success
TB: tuberculosis
TBDA: TB Drug Accelerator
